# A Keystone Gut Bacterium *Christensenella minuta*—A Potential Biotherapeutic Agent for Obesity and Associated Metabolic Diseases

**DOI:** 10.3390/foods12132485

**Published:** 2023-06-26

**Authors:** Wei-Shan Ang, Jodi Woan-Fei Law, Vengadesh Letchumanan, Kar Wai Hong, Sunny Hei Wong, Nurul Syakima Ab Mutalib, Kok-Gan Chan, Learn-Han Lee, Loh Teng-Hern Tan

**Affiliations:** 1 Novel Bacteria and Drug Discovery Research Group (NBDD), Microbiome and Bioresource Research Strength (MBRS), Jeffrey Cheah School of Medicine and Health Sciences, Monash University Malaysia, Bandar Sunway 47500, Malaysia; angweishan88@gmail.com (W.-S.A.); jodi.law1@monash.edu (J.W.-F.L.); vengadesh.letchumanan1@monash.edu (V.L.); hong.karwai@monash.edu (K.W.H.); syakima@ppukm.ukm.edu.my (N.S.A.M.); kokgan@um.edu.my (K.-G.C.); lee.learn.han@monash.edu (L.-H.L.); 2 Next-Generation Precision Medicine and Therapeutics Research Group (NMeT), Jeffrey Cheah School of Medicine and Health Sciences, Monash University Malaysia, Bandar Sunway 47500, Malaysia; 3 Pathogen Resistome Virulome and Diagnostic Research Group (PathRiD), Jeffrey Cheah School of Medicine and Health Sciences, Monash University Malaysia, Bandar Sunway 47500, Malaysia; 4 Lee Kong Chian School of Medicine, Nanyang Technological University, Singapore 308232, Singapore; sunny.wong@ntu.edu.sg; 5 UKM Medical Molecular Biology Institute (UMBI), Universiti Kebangsaan Malaysia, Kuala Lumpur 56000, Malaysia; 6 International Genome Centre, Jiangsu University, Zhenjiang 212013, China; 7 Institute of Biological Sciences, Faculty of Science, University of Malaya, Kuala Lumpur 50603, Malaysia; 8 Innovative Bioprospection Development Research Group (InBioD), Clinical School Johor Bahru, Jeffrey Cheah School of Medicine and Health Sciences, Monash University Malaysia, Johor Bahru 80100, Malaysia

**Keywords:** *Christensenella minuta*, next-generation probiotic, metabolic diseases, obesity, inflammatory bowel disease

## Abstract

A new next-generation probiotic, *Christensenella minuta* was first discovered in 2012 from healthy human stool and described under the phylum *Firmicutes*. *C. minuta* is a subdominant commensal bacterium with highly heritable properties that exhibits mutual interactions with other heritable microbiomes, and its relative abundance is positively correlated with the lean host phenotype associated with a low BMI index. It has been the subject of numerous studies, owing to its potential health benefits. This article reviews the evidence from various studies of *C. minuta* interventions using animal models for managing metabolic diseases, such as obesity, inflammatory bowel disease, and type 2 diabetes, characterized by gut microbiota dysbiosis and disruption of host metabolism. Notably, more studies have presented the complex interaction between *C. minuta* and host metabolism when it comes to metabolic health. Therefore, *C. minuta* could be a potential candidate for innovative microbiome-based biotherapy via fecal microbiota transplantation or oral administration. However, the detailed underlying mechanism of action requires further investigation.

## 1. Introduction

There has been increasing attention on the importance of gut microbiota, owing to their relationship with human health–disease conditions [1]. These rich communities of microorganisms colonize the human gastrointestinal tract. Up to 100 trillion (10^14^) microbes inhabit the gastrointestinal tract and establish a symbiotic relationship with human hosts [2,3]. The human gut microbiota is first acquired at birth and subsequently altered throughout one’s lifetime, based on a complex combination of environmental factors, dietary intake, and host genetics [4,5,6,7]. Gut microbiota plays a crucial role in maintaining host health and overall well-being. It modulates the immune response, defends against pathogens, and regulates energy balance and nutrient metabolism. However, severe chronic disease may develop when microbes are unbalanced, resulting in gut dysbiosis [8,9]. Gut dysbiosis is the disruption and imbalance of microbial diversity, the loss of beneficial bacteria, and a rise in pathogenic bacteria [10]. It has been intrinsically linked to metabolic diseases such as type 2 diabetes mellitus, obesity, and inflammatory bowel disease [11,12,13]. Therefore, maintaining a favorable equilibrium in the gut microbiome is beneficial for host health [14,15].

For the past decades, probiotics have been known as live microorganisms that confer a health benefit on the host when administered in adequate amounts [16,17]. Although most traditional probiotics, such as *Lactobacillus* and *Bifidobacterium,* are widely used, they only target the general sub-healthy population, with inconsistent therapeutic efficacy [18,19]. Recently, metagenomic approaches have been conducted in various studies to highlight the significance of commensal species in maintaining gut health and have revealed numerous new potential species, namely next-generation probiotics [20]. These new commensal species can be tailored for microbiome-based therapeutics and represent a new class of probiotics that are more effective and longer lasting than traditional probiotics. Next-generation probiotics typically resist the harsh conditions of the digestive system and colonize the gut more effectively. They produce specific compounds that offer additional health benefits, such as improved nutrient absorption and modulation of the immune system [21,22]. *Christensenella minuta* is a recently discovered bacterial species that has gained increasing attention from the scientific community, owing to its potential therapeutic applications in human health. This review discusses various aspects of *C. minuta*, including its taxonomy, distribution, and potential health benefits.

## 2. Descriptions of *Christensenella minuta*

*Christensenella minuta* is a rod-shaped bacterium with tapered ends; it is strictly anaerobic, non-motile, gram-negative, and does not form endospores. *C. minuta* is the first member of the *Christensenellaceae* family that was discovered in 2012 from 16S rRNA gene sequencing and was first cultivated from the fecal sample of a healthy Japanese male [23]. The *C. minuta* strain DSM22607 (NR 112900.1) was first reported by Morotomi et al. [24], and *Caldicoprobacter oshimai* JW/HY-331^T^, *Tindallia californiensis* DSM 14871^T^, and *Clostridium ganghwense* JCM 13193^T^ were the closest relatives, with 86.9%, 86.3%, and 86.1% pairwise IDs, respectively, based on the 16S rRNA sequence. The discovery of two novel bacterial species, *Christensenella massiliensis* and *Christensenella timonensis*, which showed 97.4% and 97.5% sequence similarities, respectively, with *C. minuta* via 16S rRNA sequencing, contributed to increased genetic divergence in the *Christensenella* genus [25,26]. In 2021, another *C. minuta* strain, DSM33407, was discovered by Mazier et al. [27], which matched a 99% sequence identity with the strain DSM22607 and displayed similar microbiological characteristics. The following year, another strain, *C. minuta* DSM 33715, was published and registered [28].

Taxonomically, *C. minuta* belongs to the phylum *Firmicutes*, the *Clostridia* class, and the *Clostridiales* order. This bacterium is named to honor Danish microbiologist Henrik Christensen, and its species name refers to its small size. (Minuta means “small” in Latin.) The genome of the strain is relatively small, consisting of approximately 1.5 million base pairs, and its DNA G+C content is 51.5 mol%. The colonies’ average dimensions are 0.507  ±  0.04 μm for width, 1.27  ±  0.28 μm for length, and 0.5–1.0 μm for diameter, occurring singly or in pairs [1]. The predominant fatty acids identified in bacterial cells are iso-C15:0, C14:0, and C16:0 [24,29]. *C. minuta* has a well-defined cell wall composed of alanine, glutamic acid, serine, and *LL*-diaminopimelic acid linked to galactose, glucose, rhamnose, and ribose as whole-cell sugars. *C. minuta* survives in an anaerobic environment and has an oxygen tolerance for at least 24 h of exposure to atmospheric oxygen. Some intestinal anaerobic colonizers have shown similar properties, such as *Bacteroides fragilis,* which remains viable after 48 h of ambient air [30]. *C. minuta* grows strongly in Gifu anaerobic medium (GAM broth) containing digested hemin serum. Other growth media used to culture *C. minuta* include reinforced clostridial medium (RCM), trypticase soy agar (TSA), brain heart infusion (BHI), and Wilkins–Chalgren anaerobic agar (WCA). *C. minuta* shows optimal growth at 37 °C and at pH 7 [27], with survivability found in medium containing <5% NaCl and 20% bile resistance for the strain DSM22607 but up to 80% bile resistance for strain DSM33715. Cells are resistant to ampicillin and tetracycline but susceptible to chloramphenicol, clindamycin, meropenem, metronidazole, moxifloxacin, and piperacillin/tazobactam [27]. *C. minuta* can utilize a variety of monosaccharides, such as glucose, D-xylose, L-arabinose, L-rhamnose, and D-mannose, to carry out saccharolytic fermentation. The major fermentation products of glucose by *C. minuta* are short-chain fatty acids, namely acetic and butyric acids, which contain no respiratory quinones. *C. minuta* shows negative results for catalase, oxidase, and urease tests. It is also incapable of reducing nitrates and cannot metabolize tryptophan [24]. Kropp et al. [31] established a metabolic model to evaluate the substrate utilization ability of the *C. minuta* strain DSM 22607. This showed that *C. minuta* can metabolize arbutin, 2′-deoxyadenosine, inosine, palatinose, salicin, turanose, fumaric acid, maltotriose, pyruvic acid, uridine, D-arabitol, D-cellobiose, dextrin, D-fructose, D-galactose, α-d-glucose, D-mannitol, D-mannose, N-acetyl-D-glucosamine, L-fucose, and L-phenylalanine [31].

As the subdominant commensal microbial species in the colon of healthy adults, *C. minuta* constitutes over 0.2% to 2% of the total bacterial population [27]. Its prevalence varies widely among individuals, with some studies reporting that *Christensenellaceae* is the most highly heritable taxon that is strongly correlated with leanness and gut metabolism. A British cohort of monozygotic and dizygotic twin studies demonstrated that the host genotype could account for 40% of the variation in the relative abundance of the family *Christensenellaceae* between individuals [27]. However, the specific human genes underlying heritability remain elusive. In the human gut microbiome, *C. minuta* forms a co-occurrence network with other heritable microbiota, namely *Methanobrevibacter smithii*, a methanogenic archaea belonging to the family of *Methanobacteriaceae* [32]. This trophic network is enriched in individuals with a lean body type, based on 16S rRNA gene-based surveys of gut microbiomes. *M. smithii*, the most abundant methanogen, produces methane (CH_4_) using H_2_ and CO_2_, the products of bacterial fermentation of dietary fibers by *C. minuta,* demonstrating H_2_ based syntrophy that correlates with a lean phenotype and a healthy status. This suggests interspecies hydrogen transfer between *C. minuta* and *M. smithii* and a positive correlation between the two species and the lean phenotype. The *Christensenellaceae* family has been proposed to act as a keystone species in the human intestinal ecosystem that facilitates the establishment of other microbial taxa [33,34]. However, further studies on these interactions are needed.

## 3. Anti-Obesity Potentials of *C. minuta* and Its Associated Metabolic Mechanisms

**Figure 1:**
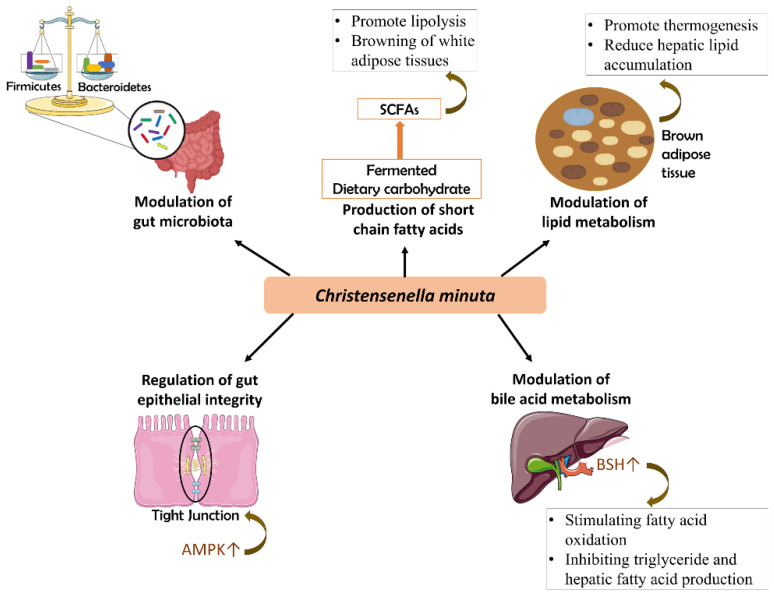
Mechanisms underlying the anti-obesity role of *Christensenella minuta.* (1) Modulation of gut microbiota—*C. minuta* renormalizes gut microbiome composition by balancing the ratio between *Firmicutes/Bacteroidetes*. (2) Production of short-chain fatty acids—*C. minuta* enhances the browning of white adipose tissues, which promotes lipolysis. (3) Modulation of lipid metabolism—*C. minuta* promotes thermogenesis and reduces hepatic lipid accumulation in brown adipose tissues. (4) Regulation of gut epithelial integrity—*C. minuta* enhances intestinal barrier integrity by regulating the tight junction assembly through the activation of AMP-activated protein kinase (AMPK). (5) Modulation of bile acid metabolism—*C. minuta* shows a high-level expression of bile salt hydrolases (BSH), resulting in the stimulation of fatty acid oxidation while inhibiting triglyceride and hepatic fatty acid production.

Obesity is a complex disease resulting from excessive body fat accumulation that can adversely affect body health. It is assessed using a body mass index (BMI) greater than or equal to 30. BMI is defined as a person’s weight in kilograms divided by the square of their height in meters [35]. According to the World Health Organization (WHO), more than one billion people worldwide are obese, with 65% of them being adults [35]. Annually, about 4.7 million premature deaths are attributed to obesity, leading to the world’s largest health problem [36]. A large range of non-communicable diseases, such as cardiovascular disease, various forms of cancer, type 2 diabetes, hypertension, and stroke, as well as mental health issues, are correlated with obesity. Recently, the etiology of obesity has been associated with dysbiosis of the gut microbiota and congenital leptin deficiency. In preclinical studies, several mechanisms have been proposed to link obesity genesis with gut microbiota composition through the dysfunction of metabolic and inflammatory activities [37]. Obesity genesis involving the gut microbiota and the host is mediated by direct interactions with proximal organs or indirect interactions with distant organs across the liver, adipose tissue, and brain via metabolite secretion [38]. In numerous studies, *C. minuta* has been repeatedly associated with its therapeutic anti-obesity potential, suggesting a strong correlation between the role of *C. minuta* in the gut microbial ecosystem and the regulation of host metabolism [34,39]. The proposed mechanisms by which *C. minuta* plays a therapeutic anti-obesity role are modulation of the gut microbiota composition, production of metabolites, lipid metabolism, gut epithelial integrity, and bile acid metabolism (Figure 1).

### 3.1. Modulation of Gut Microbiota by C. minuta

The human body comprises many microorganisms, including bacteria, fungi, viruses, archaea, and unicellular eukaryotes. Only a few bacteria phyla predominate in the human gut microbiome, namely *Firmicutes* (43.6 ± 9.2%) and *Bacteroidetes* (41.6 ± 13.1%), followed by *Verrucomicrobia* (8.5 ± 10.4%), *Proteobacteria* (2.8 ± 4.8%), *Actinobacteria* (1.8 ± 3.9%) and *Euryarchaeota* (1.4 ± 2.7%) [40]. The two major phyla, *Firmicutes* and *Bacteroidetes*, play a crucial role in maintaining gut homeostasis and preserving overall gut health. Changes in microbiota composition, called dysbiosis, particularly the abundance of specific *Firmicutes* or *Bacteroidetes* species, might lead to various pathologies. An increased or decreased *Firmicutes*/*Bacteroidetes* ratio is associated with the development of obesity and bowel inflammation, respectively [41]. Although a high *Firmicutes*/*Bacteroidetes* ratio has been characterized as a biomarker of obesity, some studies have corroborated the association between the relative abundance of *Christensenellaceae* and the host obesity phenotype, such as BMI [42]. Goodrich et al. [42] reported that *Christensenellaceae* was found in abundance in individuals with a normal BMI of 18.5 to 24.9, compared to obese individuals (BMI ≥ 30). This indicated a positive correlation between the relative abundance of *Christensenella* and leanness across populations. In addition, individuals with more weight loss had higher OTU richness (alpha diversity measure) and taxa level differences (beta diversity measure) than individuals with little weight loss, indicating the general association of overall microbiome differences with body fat percentage and total fat mass [39]. Another study showed that *C. minuta* DSM33407 treatment had a profound influence on the Shannon index (measures the diversity of species richness) of gut bacterial diversity in the colon [27]. Indeed, alteration of the *Firmicutes*/*Bacteroidetes* ratio and bacterial diversity can strongly impact the microbial composition associated with the development of digestive pathologies, such as inflammatory bowel diseases (IBD), and metabolic diseases, such as obesity. Therefore, balancing the ratio between *Firmicutes*/*Bacteroidetes* through the administration of *Christensenella* has the potential to prevent gut dysbiosis-related diseases, such as obesity and IBD.

### 3.2. Production of Short-Chain Fatty Acids by C. minuta

The most abundant metabolic products of anaerobic intestinal microbiota from the fermentation of dietary undigested carbohydrates and amino acids are short-chain fatty acids (SCFAs), primarily acetate, butyrate, and propionate, along with other fermentative gases, including carbon dioxide, methane, and hydrogen [43,44]. The abundance of acetate, butyrate, and propionate produced by bacterial fermentation was estimated at an approximate molar ratio of 60:20:20 in the cecum and colon [45]. Kropp et al. [31] found that *C. minuta* DSM 22607 produced a high level of acetate and moderate levels of butyrate at a ratio of 5:1 over three growth phases (latent, exponential, and stationary) without producing propionate. In parallel, branched-chain fatty acids, namely isobutyric acid, isovaleric acid, and isocaproic acid, were found at low levels in both the proximal and distal colons, indicating that *C. minuta* reduces bacterial proteolysis and stimulates carbohydrate fermentation. Indeed, gut microbiota is involved in the regulation of energy metabolism and gut homeostasis through balanced SCFA production [46]. SCFAs are absorbed and act as energy sources or precursors for glucose and lipid metabolism in the host. SCFAs may interact with colonic, hepatic, muscular, and adipose tissues via G-protein-coupled receptors (GPRs), namely free fatty acid receptors 2 (FFAR2) and 3 (FFAR3) [47]. Moreover, SCFAs upregulate the synthesis of the hunger-suppressing hormone leptin, inhibit lipogenesis, and promote lipolysis [48]. In the details of specific fatty acids, studies evaluated that the administration of butyrate could treat and prevent obesity by promoting adipocyte formation and browning of adipose tissue, resulting in reduced energy intake, enhanced fat oxidation, and energy expenditure. This was evidenced by profound findings on the prevention of high-fat diet-induced obesity and obesity-related disorders, such as type 2 diabetes mellitus or hypertrophy in animal models [49,50]. Some studies have shown that acetate supplementation significantly suppressed high-fat diet-induced weight gain, compared with the control group [51]. However, this is in contrast with some studies that show contradictory results [52].

### 3.3. Modulation of Lipid Metabolism by C. minuta

In addition, BMI linked with adiposity level or body fat has also shown strong associations with the abundance of *C. minuta* in the gut. The modulation of fatty acid synthesis, oxidation, and inhibition of lipogenesis favorably affects body fat and weight [39]. An earlier study demonstrated a link between the presence of *C. minuta* in the gut microbiome and lower adiposity in animal studies [42]. An interventional study with *C. minuta* showed that the accumulation of hepatic triglycerides and free fatty acids was hindered in a mouse model of diet-induced obesity. This finding was aligned with the gene expression level, where strong repression of the *gck* gene coding for hepatic glucokinase was shown in an animal model supplemented with *C. minuta* [27]. Overexpression of glucokinase facilitated excessive sugar uptake, hepatic lipid accumulation, and downregulation of thermogenic proteins in brown adipose tissue (BAT), resulting in obesity [53]. Mechanistically, enhanced adipose tissue thermogenesis in BAT and induction of the browning of white adipose tissue (WAT) could lead to weight loss [54]. In addition, the food metabolism rate was reduced without affecting feeding behavior and daily food intake. Studies have demonstrated significant weight loss and reduced adiposity gains in recipient mice that were administered with both strains of *C. minuta,* DSM22607 and DSM33407, compared to controls receiving the unsupplemented microbiome [27]. Evidence of lower fat mass and reduced hypertrophy of mesenteric WAT to anti-obesity has been obtained from animal model studies treated with *C. minuta*. A similar observation was made by Le Roy et al. [55], who reported a significant negative correlation between *Christensenellaceae* and visceral fat mass. At the hormonal level, lower circulating levels of adipokines, such as leptin and resistin, were evident in the *C. minuta* treatment group [27]. Resistin is an adipocyte-derived polypeptide secreted by adipose tissue in mice that links obesity to insulin resistance. Excessive circulating levels of leptin might induce leptin resistance, resulting in the inability of leptin to reduce food consumption and body weight [56].

### 3.4. Regulation of Gut Epithelial Integrity by C. minuta

The gut epithelial integrity is also associated with obesity. A study suggested that *C. minuta* improves the expression of intestinal tight junction proteins ZO-1, occludin (OCLN), and claudin-1 (CLDN1) *in vitro* and in animal studies involving a high-fat diet group [27]. Reduced tight junctions have been demonstrated in obese mice, which suggests that obesity results from increased intestinal permeability and decreased transepithelial resistance. Increased gut permeability has been proposed as a driving factor of fat-induced obesity, which is associated with gut dysbiosis and gut inflammation. Downregulation of intestinal tight junction proteins can lead to gut leakiness, where lipopolysaccharide bacterial substances and other inflammatory mediators diffuse through tight junctions and interact with host immune cells, resulting in low-grade inflammation, contributing to hyperphagia and an eventual gain in body weight [57,58]. *C. minuta* has been proposed to act as a membrane barrier protector during high-fat induction due to its SCFAs-producing activity [27]. In particular, butyric acid has been shown to enhance intestinal barrier integrity by regulating tight junction assembly through the activation of AMP-activated protein kinase [59].

### 3.5. Modulation of Bile Acid Metabolism by C. minuta

Bile acid metabolism is crucial for the modulation of glucose and energy metabolism, intestinal integrity, and immunity. Alterations in the bile acid metabolism are closely associated with obesity. In the context of obesity, weight loss is associated with bile acid metabolism by stimulating fatty acid oxidation and inhibiting triglyceride and hepatic fatty acid production [60]. The anti-obesity potential of *C. minuta* is exhibited, not only through the production of SCFAs, but also through the cholic acid/taurocholic acid (CA/TCA) ratio in the colon [34]. This finding is significant and provides an important theoretical basis for the association of anti-obesity with saccharolytic metabolism, as well as with the efficient deconjugation of primary bile acids. In short, primary bile acids, such as cholic acid and chenodeoxycholic acid, are converted into secondary or deconjugated bile acids, such as deoxycholic acid and lithocholic acid, by bile salt hydrolases (BSHs) because unconjugated bile salts are highly toxic to bacteria [61]. Interestingly, *C. minuta* is able to hydrolyze glycine- and taurine-conjugated bile acids, indicating that *C. minuta* is able to carry out bile detoxification and colonize human and rodent guts. Both *C. minuta* strains DSM33407 and DSM22607 are highly tolerant to bile acids in the presence of 80% bile for 48 h. The *bsh* gene has been identified in both *C. minuta* strains and is highly expressed, owing to its strong ability to hydrolyze conjugated bile acids [34]. *C. minuta* also promotes bile acid metabolism via the farnesoid x receptor and G protein-coupled bile acid receptor (TGR5), which is highly expressed in the intestine [62]. In some *in vivo* studies, the high-level expression of BSHs in the gut ecosystem has been suggested as a key regulatory factor in anti-obesity through a significant reduction in body weight, adiposity, circulating low-density lipoprotein (LDL) cholesterol, and triglycerides [60]. BSH activity is a key mechanistic target in obesity control. Enrichment of the intestinal microbiome with bacterial strains carrying high BSH activity may be a strategy for preventing and controlling obesity.

## 4. Inflammatory Bowel Diseases Associated with *C. minuta*

Inflammatory bowel diseases (IBD) is a chronic inflammatory disease that affects the gastrointestinal tract and is characterized by a dysregulated immune response in the host’s intestinal microflora [63]. There are two inflammatory conditions: Crohn’s disease and ulcerative colitis, each with different physiological symptoms. Individuals with IBD have been found to exhibit changes in microbial composition, characterized by a decrease in the *Firmicutes* to *Bacteroidetes* ratio [64]. Gut dysbiosis is accompanied by changes in SCFA composition, followed by the disruption of intestinal barrier integrity, eventually initiating inflammatory responses through immune system modulation [31,65]. Although the etiology of IBD remains poorly understood, studies suggest that they are triggered by uncontrolled inflammatory responses associated with an increase in interleukine-8 (IL-8) cytokines and reactive oxygen species (ROS). Recent *in vitro* and *in vivo* preclinical studies showed that *C. minuta* had strong anti-inflammatory and potent immunomodulatory properties. *C. minuta* reduced colonic inflammation by inhibiting the NF-κB signaling pathway and the secretion of proinflammatory cytokines IL-8 and IL-1β [28]. Moreover, the transepithelial electrical resistance ratio remained stable in Caco-2 cells treated with *C. minuta*, indicating that *C. minuta* maintained intestinal barrier integrity *in vitro*. An interventional study with *C. minuta* showed that *C. minuta* limited colon damage, promoted mucosal healing, and reduced the activation of neutrophils, specifically myeloperoxidase and eosinophil peroxidase, due to inflammation in two different animal models of acute colitis and a human intestinal cell line [31]. In addition, the concentration of LCN-2, a non-invasive biological marker of intestinal inflammation, was decreased in *C. minuta* treatment animal models. At the genetic level, individuals carrying IL23R, a Crohn’s disease risk gene, had a decreased abundance of microbes related to *Christensenellaceae,* indicating that the gut microbiome was influenced by host genetics. However, when mice were supplemented with *C. minuta*, the IL23R-protective coding variant was reported to increase, thereby protecting against Crohn’s disease [66]. *C. minuta* also produces butyrate to control the inflammatory response through the butyrate receptor, GPR109a, in adipocytes, intestinal epithelial cells, and immune cells. Remarkably, the anti-inflammatory efficiency of *C. minuta* was demonstrated to be similar to that of mesalamine, also known as 5-aminosalicylic acid (5-ASA), a medication used to treat IBD [67].

## 5. Traditional Chinese Medicine Modulates *C. minuta* for Type 2 Diabetes

Type 2 diabetes (T2D) is a complex metabolic and endocrine dysfunction characterized by hyperglycemia resulting from insulin resistance, pancreatic β-cell dysfunction, low-grade systemic inflammation, gut dysbiosis, obesity, and other endocrine disorders [68,69,70]. According to the World Health Organization (WHO), approximately 300 million people will suffer from diabetes by 2025. The prevalence of T2D is expected to be 13.5% worldwide by 2040, which presents a significant challenge to healthcare systems [71]. Traditional Chinese medicine (TCM) is another complementary medicine, derived from natural products, that possesses potential in the treatment of metabolic syndromes. Oral Chinese medicine intervention can directly impact the gut microbiome but exhibits low bioavailability, due to the poor lipophilicity properties of their active ingredients, such as flavonoids [72]. Biotransformation of the intestinal flora facilitates drug absorption, which has a significant impact on pharmacology. Moreover, TCM components may modulate the population of the host intestinal microflora. Huang-Qi-Ling-Hua-San (HQLHS), composed of *Astragalus membranaceus*, *Ganoderma lucidum*, *Inonotus obliquus*, and *Momordica charantia* L., is a specially designed Chinese medicinal formula for the treatment of T2D [73]. Recently, a study demonstrated that HQLHS inhibited pathogenic bacteria and enriched beneficial bacteria, particularly *C. minuta* and *Christensenella timonensis*, in mouse models. Notably, this study showed that HQLHS significantly increased the relative abundance of *Christensenella* in the gut flora of mice. The study also described the effect of *C. minuta* on liver metabolism, laying the basis for understanding the pharmacological mechanisms of *C. minuta* in diabetes treatment and control. In the same study, *C. minuta* DSM 22607 reduced diabetes inducers, such as oxidative stress, tryptophan, and tyrosine, in diabetic rats. The levels of antioxidant enzymes and MDA, a biomarker of lipid peroxidation, were also controlled. Several mechanisms of the anti-diabetic properties of *C. minuta* have been proposed, such as improving glycolipid metabolism, inhibiting glucose absorption by suppressing the expression of SGLT1 and GLUT2 in intestinal glucose transport, and promoting GLP-1 secretion to stimulate insulin resistance and regulate glucose homeostasis [73].

## 6. Fecal Microbiota Transplantation and Future Application of *C. minuta*

In clinical studies, fecal microbiota transplantation (FMT) has been widely implemented to resolve gut microbial dysbiosis-associated disorders, such as intestinal bowel disease, obesity, and diabetes. FMT involves the transplantation of fecal material from healthy individuals containing functional microbial communities into the patient’s gastrointestinal tract to modulate the recipient’s gut microbiome [74]. FMT has been approved by the US Food and Drug Administration (FDA) as a biotherapeutic agent to normalize gut microbiota composition in recipients [75]. Multiple studies have proven that FMT is a successful therapy for *Clostridium difficile* infection (CDI), but its efficacy remains controversial for IBD, obesity, and diabetes [76,77]. The potential risk of disease transmission associated with the gut microbiome must be considered and needs further assessment. In an earlier study, Goodrich et al. [42] demonstrated that obese mice significantly lost weight after *C. minuta* was transplanted into their gut microbiomes. This suggests that FMT of *C. minuta* is a potential therapeutic strategy for obesity and IBD. In addition, manipulation of the gut microbiome can be achieved through oral probiotic administration. The efficacy of *C. minuta* in treating obesity and its associated diseases has been reported using animal models, as presented in Table 1 [73,78].

The first clinical trial was presented by Claus et al. [79] to evaluate the safety and efficacy of *C. minuta* as a novel microbiome-based biotherapy to treat obesity and associated metabolic disorders. A new drug containing *C. minuta* DSM 33407, namely Xla1, was developed and demonstrated to be safe and efficacious in a Phase 1 clinical trial involving 28 normal-weight and obese volunteers [80]. The safety and tolerability of Xla1 in healthy adults or obese volunteers were assessed and reported to have no serious adverse events. However, treatment-related effects, including gastrointestinal disorders, were observed in the Xla1-treated and placebo-treated groups. These were common side effects of taking probiotics for the first time for the first few days as the gut microbiome underwent adaptation. Meanwhile, its efficacy was evidenced by reducing LDL cholesterol levels in obese volunteers who received daily oral treatment with Xla1 for 12 weeks [79]. This finding is significant and provides a theoretical basis for the clinical application of *C. minuta*. Nonetheless, this evidence warrants further clinical assessment, owing to the small sample size of the trial. Having said that, the researchers from Pharmabotic Research Institute and YSOPIA Bioscience shared valuable insights into their experiences and strategies in navigating the regulatory landscape, obtaining necessary authorization, and eventually advancing Xla1 as a live biotherapeutic product (LBP) into the clinical stage [81,82].

## 7. Conclusions

*Christensenella minuta* is a recently described next-generation probiotic that has received considerable attention as a new preventive and therapeutic agent. *C. minuta* displays many potential health benefits and can be a promising candidate for the treatment of various metabolic diseases, including obesity, inflammatory bowel disease, and type 2 diabetes. It plays a key role in the alleviation of metabolic disorders by regulating the intestinal microbiome. In particular, *Christensenella* can pave the way for personalized medicines or therapies based on the gut microbiome. Several studies have shown *C. minuta*’s profound anti-obesity effects through several mechanisms, including renormalization of the gut microbiome, production of functional short-chain fatty acids, inhibition of lipogenesis, maintenance of gut epithelial integrity, and regulation of energy metabolism through bile acid metabolism. Moreover, *C. minuta* alleviates inflammatory bowel disease by regulating the immune system. Interestingly, *C. minuta* is selectively promoted by TCM in alleviating type 2 diabetes through the modulation of glucose metabolism, liver metabolism, oxidative stress, inflammation, and intestinal barrier function. Interventions such as fecal microbiota transplantation (FMT) or oral administration of *C. minuta* could be effective approaches to restoring gut microbiome composition. Notably, the use of *C. minuta* as an LBP has undergone clinical investigations regarding its safety and efficacy and is currently in the pipeline for commercial development. However, it is important to note that these observed effects primarily stem from preclinical studies, which predominantly focus on animal models, with only one completed human trial. Further research is necessary to gain a comprehensive understanding of the ecological role of *C. minuta* and its interactions with other gut microbiomes. Exploring these aspects will shed light on the potential benefits of the intervention. Moreover, there is a need for in-depth studies and clinical assessments to elucidate the underlying molecular mechanisms involved.

## Figures and Tables

**Table 1:** Overview of *Christensenella minuta* intervention studies on metabolic disorders.

Diseases	Treatments	Effects	Mode of Actions	References
Obesity	2.10^9^ CFU/day of *C. minuta* cells by oral gavage for 4 to 12 weeks	Prevented HFD-induced body weight gain and hyperglycemia in diet-induced obesity mouse models; Regulated hepatic lipid metabolism by preventing intestinal lipid absorption; Regulated bile acid metabolism; Increased production of short-chain fatty acids (SCFAs); Maintained intestinal epithelial integrity.	High-fat diet-induced hypertrophy of the mesenteric WAT was significantly reduced; Plasma leptin, resistin, and delta fat mass were decreased; *gck* gene coding for glucokinase was significantly repressed; *Slc2a4*, coding for the glucose transporter GLUT4, and *Fasn*, coding for the fatty acid synthase, were regulated; Hepatic triglycerides and FFA were decreased; Ratio of unconjugated over conjugated forms of the primary bile acid cholic acid (CA/TCA) was increased; SCFAs upregulated the synthesis of the hunger-suppressing hormone leptin, inhibited lipogenesis, and promoted lipolysis; Expression of key tight junction proteins, e.g., OCLN and ZO-1, were upregulated.	[27]
	1 × 10^8^ *C. minuta* cells through FMT for 21 days	Reduced weight and adiposity gains in the *C. minuta* treatment group.	Mechanisms were not described.	[42]
IBD	10^9^ CFU/mL of *C. minuta* cells for 14 days by oral gavage	Protected from damages induced by chemically-induced colitis in mice; Induced improvement of inflammatory lesions by 36%, compared to the disease group; Possessed systemic anti-inflammatory effects.	Stimulated the production of the anti-inflammatory cytokine IL-10; Inhibited IL-8, colonic IL-1β protein, and lipocalin-2 production; Lowered macroscopic and microscopic score; Increased goblet cell number per crypt.	[28]
	*In vitro* studies Supernatant concentration of *C. minuta* culture medium (10%) *In vivo* studies Oral dose of *C. minuta* (10^9^ CFU/mL) for 2 weeks in rodents’ preclinical colitis models	*In vitro* studies Demonstrated anti-inflammatory solid activity on HT-29 cells; Maintained the integrity of the epithelial cell in the Caco-2 cell line. *In vivo* studies Reduced colonic inflammation; Induced an immunomodulatory response.	*In vitro* studies TNF-α-induced IL-8 production decreased by around 50%; NF-κB activation decreased by 40%; Stable transepithelial electrical resistance (TEER) ratio to maintain barrier integrity and limit cell damage. *In vivo* studies Concentrations of lipocalin-2 (LCN-2) and IL-1β secretion were decreased in the colon; Decreased neutrophil infiltration and myeloperoxidase (MPO) activity.	[31]
Type 2 Diabetes	1 × 10^9^ cfu/mL of *C. minuta* by intragastric administration for 6 weeks	Stimulated insulin secretion and regulated glucose homeostasis; Inhibited gluconeogenesis; Inhibited glucose transport and glucose absorption in the small intestine; Reduced oxidative stress in diabetes; Enhanced intestinal barrier; Reduced LPS-induced inflammation.	Stimulated the expression of proglucagon (*gcg*), the precursor gene encoding GLP-1, but also increased the serum GLP-1 content; Reduced hepatic expression of two gluconeogenic rate-limiting enzymes, G6PC and PEPCK; Expressions of GLUT2 and SGLT1 in ileum were suppressed; Increased CAT, SOD, and GSH-PX activity and decreased MDA serum content; Expression of colonic ZO-1 and Claudin-1 was upregulated; Hepatic and colonic expressions of TLR4, NF-κB, IL-1β, IL-6, and TNF-α were suppressed.	[73]

## Data Availability

The authors confirm that the data supporting the findings of this study are available within the article.
